# Adoption of Iran’s code of ethics for blood donation and transfusion as a public health policy

**Published:** 2019-01-22

**Authors:** Abolfazl Asghari, Aliakbar Pourfathollah, Mahmoud Abbasi, Tooran Mohammadi, Forouzan Akrami

**Affiliations:** 1 Researcher, Blood Transfusion Research Center, High Institute for Research and Education in Transfusion Medicine, Tehran, Iran.; 2 Professor, Department of Immunology, School of Medicine, Tarbiat Modares University, Tehran, Iran.; 3 Associate Professor, Medical Ethics and Law Research Center, Shahid Beheshti University of Medical Sciences, Tehran, Iran.; 4 Researcher, Department of Bioethics, Medical Ethics and Law Research Center, Shahid Beheshti University of Medical Sciences, Tehran, Iran.

**Keywords:** Ethics/morals, Blood transfusion, Donation, Public health

## Abstract

Blood is a public resource of human origin and its transfusion process is essential to individual and public health. This study aimed to develop a national code of ethics for blood donation and transfusion (BDT).

This was a qualitative research with a multi methods approach in which a combination of methods including situational analysis, focus group discussion and expert panels were used. After situational analysis and orientation, the code of ethics for BDT was developed based on the findings of a content analysis within the framework of the four principles of biomedical ethics.

The results were categorized into two sections: situational analysis and underpinnings measures, and the clauses of the code. The Iranian Blood Transfusion Organization has carried out three essential supportive measures over the past decades: approval of insurance coverage of blood recipients against communicable diseases; inclusion of 14 blood services in the book of “Relative Value Units of Health Services”; and formation of the National Ethics Committee of Transfusion Medicine. After recognition and orientation, the national code of ethics for BDT was adopted and imparted to blood donation centers. The code consists of two sections: “Blood Transfusion Centers: Donors and Donation” in 19 clauses, and “Hospitals: Patients” in 8 clauses.

The national code of ethics for BDT establishes moral norms in order to protect the rights of blood donors and recipients. It could also serve as a basis for addressing the related ethical challenges and right decision-making in the area of BDT.

## Introduction

Due to the functional necessity of establishing social relations based on common ethical values, most professions have implicitly accepted to observe a series of ethical guidelines ​​as the code of professional ethics, which is recognized by members of that profession. In recent years, guidelines have been formulated in the form of codes of ethics for medicine, nursing and research (1).

The code of ethics refers to a collection of moral norms developed in a specific profession based on a common value system. The purpose of establishing these codes is the identification and prioritization of ethical values ​​in order to guide the right action and address the ethical challenges within the profession. The criteria can be used to assess professional competence and accountability to some extent. The Hippocratic Oath is one of the first pledges that medical graduates take and are expected to observe. According to this oath, physicians should apply their utmost effort and specialty to prevent harm to patients and promote medical preservation, and refrain from unfair treatment of individuals, assistance with euthanasia and abortion, and involvement in any act of abuse, especially sexual abuse (2).

Professional moralities include ethical codes and standards for a special profession or setting, and are one form of a particular morality (1). Thus, each country establishes a code of ethics in medical areas in accordance with the human rights charter, international codes, and its own politico-moral philosophy. There is no unique model for appropriate responses against conscientious refusal that would apply to all beneficiaries and all cases; however, it has been recently emphasized that a code of ethics should contain ethical “musts” and “must nots” as well as the moral virtues in each professional area (1). Given the fact that these codes are written by experts in the profession, surveying beneficiaries and public opinions seems necessary to preserve the rights and needs of patients and the public.

Blood is a public resource of human origin, and its donation and transfusion process is essential to protection and improvement of public health (3). The special nature of blood and its components distinguishes them from other medicines and therapies, and therefore the world medical organization (WHO) has categorized them as essential medicines (4). In 1975, WHO adopted statement number 28.72 on “Utilization and Supply of Human Blood and Blood Products”, demanding that commercial activities in the private sector in the area of plasmapheresis take note of the prevailing health threats and ethical consequences, and emphasizing the necessity of establishing regulations to protect the health of donors. This statement asked member governments to develop blood transfusion (BT) on a voluntary and non-remunerated basis before discussing the issue of the code of ethics for blood donation and transfusion (BDT). There were serious concerns about concentrating exclusively on the production and provision of more blood and blood components and disregarding the threats to the health of donors and the quality and safety of donated blood. Consequently, WHO and the International Society of Blood Transfusion (ISBT) worked together to develop the code of ethics for BT to address these concerns and regulate the relationship between blood transfusion centers, blood donation volunteers and patients based on the mutual rights and obligations specified by ISBT. The above-mentioned code was adopted by the general assembly in 1980 (5) and updated in 2000 with an emphasis on the importance of voluntary non-remunerated blood donation, and was later amended by WHO and Red Cross associations on September 5, 2006 (6). 

In the years 2005 and 2010, WHO adopted two statements on the observation of the rights of blood donors and recipients: 1) Blood Safety: Proposal to Establish World Blood Donor Day (No: 58.13), and 2) Availability, Safety and Quality of Blood Products (No: 63.12) (7). Accordingly, the January 2017 report shows that the organization has established a framework in terms of ethical rules as well as structural and procedural requirements for human-originated medical products with the aim to write a comprehensive and integrated document, which was published after a widespread survey (8). As a basis for the health of donated blood, quality assurance cannot bring the risks to zero (9, 10); however, due to the inherent risks of BT, the code of ethics for BT can be considered as a guarantee for donation and health of the donated blood in functional terms. 

The Iranian Blood Transfusion Organization (IBTO) was established in May 1974 with the main goal of providing safe blood and blood components. The safety and adequacy of blood supply depends on well-managed blood donation and distribution by the steward organization. The key strategy to provision of adequate and safe blood supply is recruitment and retention of voluntary and non-remunerated blood donors (11). Although blood donation in an Iranian-Islamic context is considered as a charitable and ethical act, it is necessary to raise awareness among community members and encourage them to continuously donate blood. A 2009 study of the knowledge, attitudes and practices of the Iranian population on the subject of blood donation showed that 55% of the donors had donated blood more than once. The most common motivation for blood donation was altruism, and the most important obstacle was the difficulty of access to donation centers (12). On the other hand, the commitment of the steward organization to support the right to health care requires enhanced community participation and the trust of blood donors. By developing and enacting the code of ethics, the IBTO can increase the supply of safe blood through reassuring the public, resolving their concerns and doubts, and establishing good services. 

Thus, considering the politico-moral foundations of the country’s health care system, including the right to health care and the obligation of the state to provide it, and observation of distributive justice with a focus on vulnerable and disadvantaged groups (13), the present study aimed to develop the national code of ethics for BDT.

## Method

In this qualitative study, the multi-method approach was used, which is a combination of methods that produce data of the same type (14). This approach brings about integrity of the findings and increases the credibility of the research (14, 15). In the present study, the data obtained at different stages of the study were mainly of qualitative type. Establishment of the code of ethics for BDT in Iran began in December 2015 in response to the proposal of the director of the IBTO to the legal department of the organization. 

In the first stage, analysis of the current situation was performed through an investigation of relevant publications using Mesh subheadings of “blood” [MeSH Terms] and “ethics” [MeSH Terms] in PubMed database; Non-Mesh subheadings of “ethics” and “blood donation and transfusion” via Google Scholar search engine; and the IBTO website for national documents. At the next stage, a focus group discussion was held to receive opinions about the themes to be considered in the drafting of the code as well as the main ethical issues in BDT. The focus group consisted of 14 expert specialists in the fields of medical ethics (2 persons), medical law (2 persons), medicine (2 persons), pharmacy (2 persons) and organizational management (6 persons). These people were selected purposefully by the supreme council of IBTO. After explaining the purpose of the study and obtaining verbal consent of the participants, formal invitation letters were sent to them. The group discussion was conducted and recorded by the first researcher and lasted 1 hour. The collected data were analyzed using deductive content analysis under the two main categories of Blood Transfusion Centers: Donors and Donation, and Hospitals: Patients

Considering the international code of ethics for BT and the findings of the group discussion, the national code of ethics for BDT was drafted and codified within the framework of principlism. The primary draft was discussed and completed in two expert panels with the contribution of 12 member persons of the Supreme Council of IBTO, including experts in the fields of blood transfusion medicine (3 persons), medical ethics (2 persons), medical law (1 person), immunology (1 person), hematology (1 person), pharmacy (1 person), pathology (1 person), community medicine (1 person), and Food and Drug Administration (1 person). The second draft was then sent as an official letter to 10 managers of the provincial bureau of blood transfusion, 3 specialists of blood transfusion medicine, and 3 specialists of medical ethics who were not employed in the IBTO for external review. Simultaneously, the comments of 6 community members who were familiar with blood transfusion concepts were also collected. After receiving the comments and applying them, the third draft was submitted to the department of quality assurance and control as well as the deputy of technics and new technologies of the IBTO in order to receive their approval. The last draft was then propounded and finalized in the meeting of the ethics council on transfusion medicine in February 2017, and was adopted and communicated to blood transfusion centers throughout the country. A number of the clauses in this code refer to the performance of hospitals regarding the transfusion of blood and its components, and the way in which patients use such services. Therefore, in October 2017, the national code of ethics was discussed and approved with minor revisions in a meeting with representatives of the main stakeholder organizations including the Ministry of Health and Medical Education, Medical Council, and Council of Ethics of the Iranian Academy of Medical Sciences.

Selection of the participants in discussion sessions was based on the objectives of the study. All research steps were in accordance with the national code of ethics in biomedical research. Verbal consent was obtained from the participants to attend discussion sessions, and official invitation letters were sent to them before each session.

## Results

The results are categorized in two sections: the current situation and underpinning measures, and the code of ethics for BDT. 

***A) The current situation and underpinning measures***

IBTO is a non-profit organization affiliated to the Ministry of Health and Medical Education and operated by the supreme council, which is composed of five expert members selected by the minister of health as the decision maker and managing director as the pillar of implementation. According to article 4 of the statute of the IBTO, adopted in 1984, the supply, provision and distribution of blood should be entirely free of charge. In the case of blood products and derivatives that require specialized and costly processes, according to article 7, clause (c) of the statute, the cost of the added processes can be received based on the tariff approved by the supreme council of IBTO. According to the organization’s statute, blood transfusion services such as the recall and recruitment of volunteers for donating blood and cellular and plasma products, the selection of donors from among these volunteers, blood sampling and screening, and preparation and distribution of the blood and blood components to the hospitals is exclusively the responsibility of IBTO. In this regard, the prescription and transfusion of blood and blood components is beyond the responsibility of the organization and falls within the expertise of responsible physicians. However, article 7, clause (d) of the organization’s statute is merely implemented in hospitals through the supervision of the IBTO according to the “Code of Activity of the Blood Bank and Blood Consuming Sectors” as one of the requirements for ensuring the health of the blood cycle. All hospitals have a structure called the BT section, which operates as blood bank and blood transfusion center and management. The BT department and the physician in charge collaborate to manage the blood transfusion cycle, since the need is announced for the blood and blood components until they are administered and the patient’s state is preserved.

One document that is observed in the records of the IBTO is the “Ethics Charter”, in which the seventh clause and its following four sub-clauses refer to the ethical principles of blood donation. This clause comprises issues such as voluntary donation, obtaining the written consent of volunteers, preserving donors’ health, the necessity of donors’ awareness of their ethical responsibility toward recipients of blood and the possible risks of transmitting infections, and the confidentiality of donor information.

The IBTO has adopted the following measures in the past decade, which can be considered as supportive actions for donors and recipients of blood transfusion services: First, based on the proposal of the IBTO and the approval of the Islamic Consultative Assembly of Iran in 2007, recipients of blood transfusion services were covered by insurance companies against the possible complications of blood consumption. In the following years, the IBTO provided insurance for not only recipients, but also blood donors against such probable events. Second, fourteen cases of blood transfusion services were mentioned in the second edition of the book titled “Relative Value Units of Health Services” since 2015 through approval of the supreme council of health insurance and approval of the cabinet of ministers, and these services are currently covered by social security. Third, the “Ethics Committee of Blood Transfusion Medicine” was established with the aim of determining the policies and strategies related to the ethics of BDT and decision making about the ethical challenges in this area, as well as developing and supporting education and research activities in terms of the ethical considerations in BDT. Finally, formulation of a code of ethics for BDT was included in the IBTO agenda. 

***B) Code of Ethics for Blood Donation and Transfusion***

Using deductive content analysis, the following themes emerged under two main categories: 

*1) Blood Transfusion Centers*: *Donors and Donation *

In this category 8 themes were extracted: voluntary non-remunerated blood donation; respect for the rights of donation volunteers; continuity of donation and assurance of the safety of blood supply; requirements for maintaining safety of the blood transfusion cycle from donation to injection; commitments and obligations of blood transfusion centers; privacy and confidentiality; exemptions; and education and research. 

*2) Hospitals: Patients*

The following 4 themes emerged in this category: protecting the rights of blood recipients; responsibilities and obligations of blood transfusion centers; observing clinical standards; and managing blood consumption. 

The code of ethics for BDT was formulated and adopted based on the obtained subthemes and in the framework of the four principles of beneficence, non-maleficence, respect for autonomy and justice. The code included an introduction and the main body, which consisted of two parts: “Blood Transfusion Centers: Donors and Donation” in 19 clauses, and “Hospitals: Patients” in 8 Clauses (Appendix).

*Blood Transfusion Centers: Donors and Donation*

The first clause of section 1 emphasizes voluntary, non-remunerated blood donation, done only for the purpose of helping one another and with altruism as motivation. Thus, volunteers donate their blood freely without expecting any type of reimbursement such as cash or cash alternatives. On this basis, even the granting of a work leave is recognized as a form of wage, unless it is necessary for the referral and conduct of the donation process. However, entertaining donors or reimbursement of transportation costs is acceptable when necessary. 

Clauses 2, 3 and 4 concern the informed consent of donors and related issues. Clause 5 emphasizes donors’ awareness of the consequences and risks of donating blood and their ethical responsibility toward the recipients of donated blood. According to clause 6, donors must answer the questions that are asked of them honestly and accurately. Clause 7 points out that blood transfusion centers are obliged to inform donors about cases of exemption from blood donation as well as the reasons, and if the volunteer is exempted, the center should declare the cause, type and duration of the exemption. Clause 8 emphasizes that care and recommendations after blood donation should be clearly explained to volunteers/ donors, and this should be confirmed by them. Clause 9 clarifies that donors can cancel the blood donation process at any stage. Clauses 10 and 11 emphasize the necessity of respecting the privacy of volunteers/donors and the confidentiality of their information, and states that they should be aware of these considerations. Clause 12 emphasizes that blood donors and recipients should not be aware of one another’s identity, barring exceptional cases.

Clauses 13 and 14 point out the necessity of observing the national BT standards and state that all BT processes should be carried out under supervision of a qualified physician. Clauses 15 and 19, respectively, refer to non-discrimination on the grounds of gender, race and socio-economic status, and fair access for all patients. Clause 16 states that blood donors and recipients should be aware of any complications, and clauses 17 and 18 emphasize that the organization should avoid profitable incentives and develop executive processes to prevent the loss of this public resource and protect individual and public health.

*Hospitals: Patients*

In this section, in addition to the emphasis on patients’ best interests, clause 1 highlights the right of patients to be aware of the risks and benefits of transfusion and alternative therapies. Moreover, clause 2 states that transfusion of blood and blood components should only be conducted with the informed consent of the patients, their parents or legal representatives. Based on clause 3, the patient can cancel the process of receiving blood at any stage of the treatment. To make this decision, the patient should be aware of the potential consequences, and the decision should not affect the behavior and function of the hospitals and their staff. In clause 4, based on the charter of safety of blood and its components, all relevant processes in the treatment centers must be in accordance with national BT standards. According to clause 5, the only basis for the administration of blood transfusion is the clinical condition of the patient, and in cases where the patient needs only a particular part of blood (for instance plasma or cell derivatives), prescribing the whole blood is not allowed. Clause 6 states that prescribing blood or blood components should not be based on financial incentives or benefits. Clause 7 specifies that adoption of measures such as the use of blood substitutes, autologous transfusion, limited use of blood transfusion, and optimal blood management are the professional obligations of medical practitioners. Finally, according to clause 8, the administration and transfusion of blood and/or blood components should be carried out under the supervision of qualified physicians.

## Discussion

The main goal of the IBTO is to provide universal access to sufficient and safe blood and blood products and ensure their appropriate clinical consumption. The situation analysis indicates the necessity to establish the national ethics committee of transfusion medicine and develop the main infrastructures, including regulations on the provision of safe and sufficient blood supply, by the supreme council of IBTO over the past decade (16). Leadership and governance is one of the main domains that has been emphasized and assessed by the WHO-Eastern Mediterranean Region. The assessment report of blood transfusion services showed that among 18 respondent countries, 14 have a national policy, 13 have strategic plans, 12 have advisory committees, and only 9 countries have a legislative mechanism (17).

Based on the “framework for action for blood safety and availability 2016 - 2025” (17), the second phase of the present study was performed to develop and implement a legislative system for supervising the blood donation and transfusion services. Accordingly, the national code of ethics for BDT was formulated and codified based on scientific standards and the four principles of biomedical ethics and its derivatives, including: consideration of the benefits and burdens of patients and donors; respect for autonomy and the rights of donors and patients; emphasis on respect for privacy and confidentiality of information; and equity in two sections: “Blood Transfusion Centers: Donors and Donations” and “Hospitals: Patients”. Due to the nature of blood and blood donation as a public resource, the first section of the national code was mostly formulated based on the principles of public health ethics (18, 19). The second section, however, was mainly focused on the principles of clinical ethics due to its patient-centered nature (20). For this reason, in the second section of the national code, all the terms of the international code have been addressed and the obligations of health professionals according to clinical and para-clinical guidelines have been emphasized.

The code of ethics of ISBT has also been divided into two sections: “Blood Transfusion Centers: Donors and Donations” in 11 clauses, and “Hospitals: Patients” in 7 clauses. The pillars of this code are the following ethical elements (21): the principle of voluntary non-remunerated blood donation without material incentives as the basis for blood transfusion services, and universal access to blood as a public resource. A review of the 2017 version of the international code indicates that the revision has been based on human dignity and the four principles of biomedical ethics, which is similar to the national code developed in this study. The difference in the number of clauses in the first section is due to the emphasis on instances of informed consent and the ethical responsibility of volunteers for public health in the national code. 

Access to blood mainly relies upon voluntary and non-remunerated contribution of a human donor (22). Thus, the first clause of section 1 emphasizes voluntary and non-remunerated blood donation with the motive of altruism. A survey conducted in Shiraz Blood Transfusion Center on motivations for blood donation showed altruistic causes to be the main motive at 65.3%, while other common motivations included controlling individual health status, earning money, treating illnesses and curiosity in that order (23). According to the regional strategic framework for blood safety and availability (2016 - 2025), the volunteers/donors and their actions must be respected, and all necessary measures should be taken to protect their health and safety, and to ensure that blood products are equitably and appropriately distributed (17). Therefore, given the importance of protecting donors as the main source of blood supply in continuous voluntary non-remunerated blood donation, the next clauses mainly focuse on respecting the rights of the volunteers/donors. 

One of the 10 basic functions of public health is mobilizing the community to contribute to solving health problems (24). A sense of social responsibility will lead to participation in public health activities to achieve public good. According to Weed and McKeown et al. responsibility is a profound concept that is linked to commitment to someone or something. Therefore, being responsible entails commitment to a positive action, or the attempt to achieve a high value such as social or public good (25). 

In public health, social responsibility has been considered as a moral norm, which, in addition to promoting participation, helps to protect and promote public health (26). In this area, people are responsible for what will happen if preventive measures are not applied, which is referred to as consequentialist responsibility (27). With regard to blood donation for check-up purposes, especially by donors with high-risk behaviors, clauses 5 and 6 emphasize the moral agency of the donors and confidential self-exclusion strategy to ensure the health of both donors and recipients (28). In terms of accountability (17), the focus of these clauses is on the proper governance and management of the blood transfusion cycle. As previously mentioned above, the second section of the code is mainly patient-centered; clauses 1, 2 and 3 of this section pertain to respecting the rights of recipients and patients, while the succeeding clauses are about observing professional standards and patient blood management. Promotion of the clinical use of blood and blood products (17) and patient blood management are priority interventions to optimize the care and improve patient safety and clinical outcomes (29, 30). 

In the next phase of the study, the revision of the code will be considered 3 years after adoption. For this purpose, the new national evidences and recommendations of the main stakeholders will be investigated with more focus on the moral norms in public health policy and the stewardship role of the IBTO. 

## Conclusion

The developed national code of ethics for BDT adopts moral norms for protecting the rights of blood donors and patients alike. It uses human rights literature and the values ​​of the Iranian health care system and transnational documents within the framework of citizenship laws. These norms, along with cultural beliefs and values, develop a common ethical framework in the relationships among volunteers/donors, blood transfusion centers, health centers and hospitals, medical doctors and other health professionals, and the patients; at the same time, they provide a basis for addressing ethical challenges and making the right decisions in the area of BDT. Since these moral norms have been formulated based on respect for the rights of volunteers and donors, they can encourage volunteers to continue donation and can be effective in supplying adequate and safe blood supplies. The internalization of these moral norms as well as their observation as an organizational behavior by medical professionals and blood donors can ensure the implementation of the code. 

## Appendix

Iran’s Code of Ethics for Blood Donation and Transfusion

Purpose 

The purpose of the code is to establish ethical and professional principles that should be observed in all activities in the field of blood donation and transfusion according to the Iranian Blood Transfusion Organization (IBTO). 

Introduction

The code of ethics for blood transfusion and donation puts forward a set of moral norms to protect the rights of blood donors and patients. These norms, along with cultural beliefs and values, establish a common moral system for blood donors, blood transfusion centers, medical centers, medical practitioners and patients, while establishing a basis for addressing ethical challenges and making related decisions and solutions. The main foundations for developing the code included: observing scientific and ethical principles, considering donors’ and patients’ interests as well as harms that they may suffer, emphasizing the privacy and confidentiality of donors’ information, respecting the dignity and rights of patients and donors, and obtaining informed consent.

Definitions

“*Blood Donation*” is the voluntarily donation of blood, which is collected either for direct transfusion or preparation of a medicinal product for human use. 

***“*** *Blood Transfusion Center* ***” ***refers to an entity that is responsible for any aspect of the recruitment of donors, collection and screening of blood, and also blood processing, storage, and distribution, when intended for transfusion. 

***“*** *Screening* ***”*** refers to a laboratory process that determines the safety of donated blood by performing tests to detect certain infectious agents transmitted through the bloodstream.

***“*** *Qualified Physician* ***” ***is a person who holds a valid medical professional license and has passed related training courses and received a certificate.

***“*** *Autologous Transfusion* ***”*** is a condition in which donated blood is used exclusively for self-administration by the donor.

***“*** *Restrictive Transfusion Strategy* ***”*** is blood transfusion that is performed only if the patient has a hemoglobin level of less than 7 g/dl and is considered in stable condition. 

***“*** *Patient Blood Management* ***”*** is an evidence-based, patient-centered and multidisciplinary approach that is adopted to manage anemia, minimize iatrogenic blood loss, and harness tolerance of anemia in an effort to improve patient outcome.

Blood Transfusion Centers: Donors and Donation

1. Blood donation must be voluntary and non-remunerated under all circumstances and performed solely for the purpose of altruism. Volunteers donate their blood without any compensation, whether cash or cash alternatives. Thus, even a work time off, other than for the purpose of conducting the donation process, is recognized as a form of wage. However, refreshments and reimbursement of direct travel costs are not contrary to voluntary, non-remunerated donation.
2. Blood donation must be voluntary and done with the donors’ informed consent. Donors have the right to be clearly informed about the conditions for donating blood, the stages, and any actions and expenditures related to blood and blood components, and their consent should also be obtained to use the blood and blood components.
3. Donors have the right to be fully informed about the blood donation conditions and processes.
4. Donors have the right to be informed about the potential risks of blood donation. In all cases, the priority is to protect the health and safety of the donor.
5. Donors should be aware of the hazards and consequences of donating infected blood, and of their moral responsibility toward potential recipients.
6. Donors should answer the questions honestly and clearly.
7. Blood transfusion centers must inform donors about cases of exemption from blood donation and the reasons. In the event of a voluntary exemption from donation or detection of infections during the screening process, the centers should inform donors about the type, cause and duration of exemption.
8. Post-donation care should be clearly communicated and it must be established that the donor has fully understood it.
9. Donors can cancel the blood donation at any stage.
10. The privacy of donors must be respected during the medical interview and the screening process.
11. The information of donors must not be disclosed, and they must be ensured about the confidentiality of their information and medical records.
12. Blood donors and recipients should not be aware of one another’s identity, barring exceptional cases. Blood transfusion centers and hospitals must take all the necessary measures in this respect.
13. All the processes related to blood donation must be performed in accordance with the standards of the National Blood Transfusion Organization, and under the supervision of a qualified physician.
14. The only criterion for accepting or exempting donors is the standards of the National Blood Transfusion Organization. Physical examinations and interviews must be conducted under the supervision of a qualified physician.
15. Any discrimination based on gender, religion, ethnicity or nationality is prohibited. Blood donation centers and their staff should not make discriminatory requests from donors and recipients.
16. Blood donors and recipients must be informed about any possible complications.
17. Establishing blood transfusion centers and providing blood services must not be done for commercial motives and profitable purposes.
18. All measures from donation up to blood transfusion should be implemented in such a way as to prevent the loss of blood and blood components.
19. All patients must have access to blood and blood components when necessary.

Hospitals: Patients

1. Patients have the right to be clearly informed about the risks and benefits of blood transfusion as well as alternative therapeutic procedures by the physician.
2. The transfusion of blood and its components must only be performed after obtaining the written informed consent of the patient or her/his legal representative; if this is not possible, the decision for transfusion of blood and blood components should be based on the “best interests of the patient”.
3. The patient has the right to refrain from receiving blood or blood components at any stage of the treatment. In this case, he/she should be notified of the possible consequences of the decision, and the final decision should be documented and approved by him/her. However, such a decision should not adversely affect the behavior of physicians and medical practitioners in providing the best alternative treatment and reducing the consequences of the patient’s decision.
4. To ensure the safety of the blood and blood components, all relevant processes must be done in accordance with the standards of the National Blood Transfusion Organization.
5. The blood transfusion must be done only based on the patient’s clinical condition; therefore, in cases where the patient only needs a specific component of the blood (plasma or cell derivatives), administration of the whole blood is not permitted.
6. The administration of blood or blood components must not be due to financial incentives.
7. Adoption of measures such as the use of blood substitutes, autologous transfusion, restrictive transfusion strategy and patient blood management fall within the domain of professional ethics, and must be supervised by responsible physicians.
8. The prescription and administration of blood and blood components must be done by a qualified physician.

- Blood transfusion centers, hospitals, and other related centers are required to comply with this code of ethics and take it into account when compiling or revising their guidelines.
- The ethics committee of the Supreme Council of IBTO is responsible for the supervision and revision of the code.

## Notes:

**Citation to this article:**

Asghari A, Pourfathollah A, Abbasi M, Mohammadi T, Akrami F. Adoption of Iran’s code of ethics for blood donation and transfusion as a public health policy. J Med Ethics Hist. 2019; 12: 1.
